# Management of hyperthyroidism in pregnancy

**Published:** 2008-11-15

**Authors:** C Grigoriu, C Cezar, M Grigoras, I Horhoianu, C Parau, P Virtej, A Lungu, V Horhoianu, C Poiana

**Affiliations:** * Obstetrics–Gynecology Clinic – University Emergency Hospital BucharestRomania; ** Endocrinology Institute C. I. ParhonRomania; *** Medical University Carol Davila BucharestRomania

**Keywords:** hyperthyroidism, pregnancy

## Abstract

Maternal hypertiroidism is a relative rare disorder, which can seriously complicate pregnancy in each of its periods. There are several maternal and fetal complications during pregnancy, delivery and postpartum period. Correct management includes an accurate diagnosis, rigorous individualized treatment and minutious follow–up. We are presenting a retrospective study of 38 pregnant women who delivered in the Obstetric Unit of the University Emergency Bucharest Hospital in the past five years. We established a follow–up protocol in collaboration with endocrinologists. Precocious diagnosis of pregnancy is, in our opinion, mandatory. Accurate diagnosis of hormonal status beginning from the first week of pregnancy is of great importance. Maternal (weight, BP, TSH, thyroid hormones, ECG, etc.) and fetal (ultrasound, non–stress test, Doppler study) evaluation during pregnancy were rigorous performed. Results: abortion rate was 5%; 15% of pregnant women delivered prematurely; cesarean section rate was 22%; fetal outcome was excellent. Treatment adjustment during pregnancy was frequent, 28% of pregnant women had no hormonal treatment in the last trimester of pregnancy. Maternal complications were rare (poor weight gain, tachycardia). Fetal complications included low birth weight (24%), fetal respiratory distress (10%). Conclusions: team work with experienced endocrinologists and understanding of versatility of disease leads to good prognosis of mother and fetus in presence of hypertiroidism.

## Introduction

Maternal hyperthyroidism is a relative rare disorder, which can seriously complicate pregnancy in each of its periods. The most common cause of hyperthyroidism during pregnancy is Graves' disease [1]. Graves' disease is a complex autoimmune disorder, characterized by autoantibodies that activate the TSH receptor. These autoantibodies cross the placenta and can cause fetal and neonatal thyroid dysfunction even when the mother herself is in an euthyroid condition. Exceptional, hyperthyroidism in pregnancy has a different cause other than Graves' disease like hyperemesis gravidarum, gestational transient hyperthyroidism, hydatiform mola, choriocarcinoma [1, 2].

## Clinical consideration and diagnosis

The signs and symptoms of hyperthyroidism can include tachycardia, palpitations, heat intolerance, nervousness, goiter, weight loss, thyromegaly, exophthalmia, increased appetite, nausea and vomiting, sweating, and tremor. Many of these symptoms are also seen in pregnant women who have normal thyroid function. The most discriminatory features of hyperthyroidism in pregnancy are persistent tachycardia, weight loss, systolic flow murmurs, tremor, lid lag and exophthalmia. 

Most pregnant women with hyperthyroidism have already been diagnosed prior to pregnancy. 

The diagnosis of overt hyperthyroidism rests on laboratory tests, particularly on the estimation of suppressed serum TSH. There are also elevated levels of free thyroxin (FT_4_) and free triiodothyronine (FT_3_). Subclinical hyperthyroidism is defined as a suppressed TSH level with normal FT_4_ and FT_3_ levels. A form of hyperthyroidism called the T_3_– toxicosis syndrome is diagnosed by suppressed TSH, normal FT_4_, and elevated FT_3_ levels [2, 3]. 

Pregnant women tolerate mild to moderate degrees of hyperthyroidism relatively well. If the diagnosis is in doubt, the thyroid function tests can be repeated in 3 or 4 weeks before making a final decision.

Graves' disease, being an autoimmune disease, may be exacerbated in the early parts of pregnancy, but as immune suppression typically occurs with the pregnancy, Graves' disease improves. Postpartum, the patient may remain in a permanent remission, but recurrence is also possible.

## Measurement of antibodies

Antithyroid antibodies are common in patients with autoimmune thyroid disease, as a response to thyroid antigens. The two most common antithyroid antibodies are thyroglobulin and thyroid peroxidase (anti–TPO). Anti–TPO antibodies are associated with postpartum thyroiditis and fetal and neonatal hyperthyroidism [4]. TSH–receptor antibodies include thyroid–stimulating immunoglobulin (TSI) and TSH–receptor antibody. TSI is associated with Graves' disease. TSH–receptor antibody is associated with fetal goiter, congenital hypothyroidism, and chronic thyroiditis without goiter.

Recent studies investigated the relationship between the presence of antithyroid antibodies and pregnancy complications, finding a high proportion of women with previous history of obstetric complications and high levels of circulating anti–thyroid peroxidase antibodies and anti– thyroglobulin antibodies. Furthermore, thyroid function disorders may affect the course of pregnancy [5]. 

Antibody patterns generally fluctuate with pregnancy, reflecting the clinical progress of the disease, but may remain stable in patients with low antibody titers [6]. TRAbs can be detected in the first trimester, but values often decrease during the second and third trimesters and might become undetectable before increasing again postpartum [5, 6]. Clinically, patients can experience relapse or worsening of Graves' disease by 10–15 weeks of gestation. Graves' disease can, however, remit late in the second and third trimesters.

The antibodies should be measured in the following situations:

- Women with Graves' disease who had fetal or neonatal hyperthyroidism in a previous pregnancy
- Women with Graves' disease who receive antithyroid drugs
- Euthyroid pregnant woman with fetal tachycardia or intrauterine growth restriction
- Presence of fetal goiter on ultrasound.

## Maternal–fetal complications

Hyperthyroidism can have multiple effects on the pregnant woman and her fetus, varying in severity from the minimal to the catastrophic.

If left untreated, and it should not be left untreated, it is possible to have hypertension, congestive heart failure, thyroid storm with labor, increased abortion rate, premature labor, stillbirth or neonatal death, low birth weight baby, fetal abnormalities. 

## Material and methods

We performed a retrospective study of 38 pregnant women, who were treated and delivered in our clinic in the past five years. The most common cause of hyperthyroidism was Graves' disease (34 patients). Other causes were hyperemesis gravidarum and gestational transient hyperthyroidism. 

The most common clinical signs were: palpitations, tachycardia, heat intolerance and emotional liability. Discriminatory features were persistent tachycardia, weight loss, systolic flow murmur, and tremor.

Together with endocrinologists, we established a follow–up protocol. Accurate and precocious diagnostic of hormonal status beginning from the first weeks of pregnancy is of great importance.

Our protocol included preconceptional counseling, antenatal, intrapartum and postnatal management, with careful maternal and fetal evaluation [7].

### Preconceptional counseling

Preconceptional advice is very important. We believe that counseling about the effects of the disease on maternal health and on fetal well– being remove anxiety commonly present among these women. The patient's thyroid status should be checked frequently to minimize risk of miscarriage [8].

We informed the patients that discontinuing the treatment would lead to a higher risk of mortality and morbidity to both the mother and her fetus. In 2 cases, where the treatment was initiated with methimazole, we changed to propylthiouracil and we ensured the patients that the medication is not teratogenic and safe to be used in pregnancy [7, 8].

### Antenatal and intrapartum management – previously treated patients with Grave's disease

In our group, 20 patients received antithyroid drugs and 4 of them underwent surgery. The important concern here is that fetal and neonatal hyperthyroidism may still occur.

According to recent guidelines, the measurement of the TSH receptor antibodies is not required, if antithyroid drugs have been used because the maternal thyroid function provides a reliable estimate of fetal thyroid status and the risk of neonatal hyperthyroidism is very low.

The TSH receptor antibodies will be measured in an euthyroid pregnant woman previously treated by radioiodine therapy or surgical. If the level is high, the fetus should be carefully assessed during gestation and the antibodies measured again in the last trimester [9].

**Table 1:** Guidelines for measurements of TSH – receptor antibodies in a pregnant woman with Grave's disease (9)

Patient status	Antibodies measurement
Euthyroid – previous antithyroid drugs	Not necessary
Euthyroid ± T4 therapy and Previous radioiodine therapy/surgery	Check in early pregnancy:if low or absent no further testing or if high: check fetus and check antibodies in last trimester
Receiving antithyroid drugs during pregnancy	Measure in last trimester

In our series, the antibodies in women who underwent surgery were very low [7].

###  Antenatal and intrapartum management – patients found to have Grave's disease in early pregnancy

We preferred medical therapy as radioiodine is contraindicated and surgery requires pretreatment with antithyroid drugs to render the patient euthyroid.

**Thioamide drug therapy (propylthiouracil, methimazole, carbimazole)** is the first line therapy, indicated for moderate or severe hyperthyroidism. 

The drug of choice is propylthiouracil, given in a dose of 100–150 mg three times daily until the patient becomes euthyroid (with normal thyroid function tests) at which time the dose should be reduced to the lowest amount to maintain the euthyroid state.

Although there have been no prospective clinical trials, multiple case reports have associated methimazole with two types of fetal abnormalities: choanal or esophageal atresia and aplasia cutis [10].

**Beta–blockers** are relatively contraindicated, but not absolutely, so that propranolol can be used until T4 levels normalized.

The complications of the drugs include:

- Lower Apgar scores
- Intrauterine growth retardation
- Postnatal bradycardia, hypothermia and hypoglycemia
- Neonatal respiratory distress [9, 10].

Our series is characterized by a multidisciplinary effort between family physician, obstetrician and endocrinologist, in order to achieve optimal control of hyperthyroidism.

Most of the patients (82%) received medical therapy, based on thioamides as first line therapy and propranolol in 2 cases. The use of propranolol was associated with releasing of sympathetic symptoms like tachycardia, tremor and sweating. We used a dose of 30 mg for short periods and after that we reduced it gradually.

28% of patients had no hormonal treatment in the last trimester of pregnancy, being very careful followed–up.

Intrapartum, we maintained the lowest dose of medication. 

Vaginal delivery was the mode of choice (70%) and caesarean section was reserved for obstetrical indication (22%).

We present the algorithm we used in our clinic (1) [7].

After an accurate and as possible precocious diagnosis, we started antithyroid drugs, until the patient became euthiroid. We continued with the lowest dose of antithyroid drugs up and during labor.

The thyroid function was monitored every 4 weeks; with adjust of medication, when necessary.

We checked the antibodies in the third trimester, at 36 weeks.

Counseling of the patient was also important; we discussed the maternal–fetal and breastfeeding effects of the treatment.

It is also important to check the infants for possible thyroid dysfunction.

We reviewed the patients postpartum for exacerbation.

**Fig 1:**
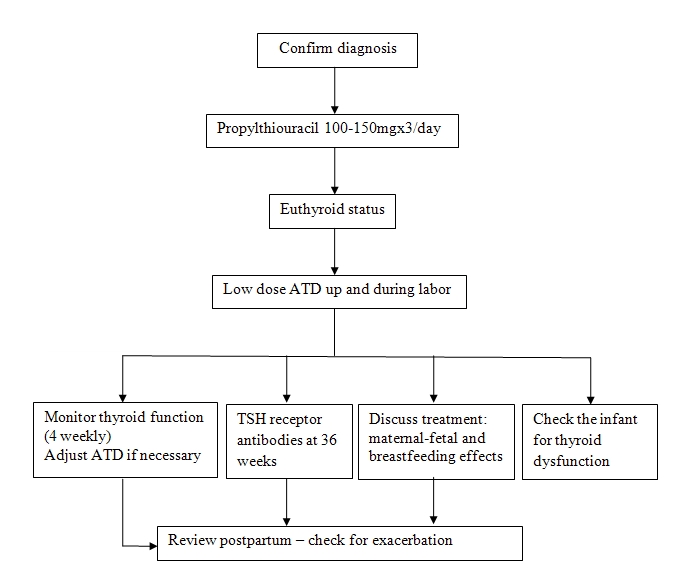
Management of hyperthyroidism in pregnancy (7)

### Postpartum management

During the postpartum period, the mother should be encouraged to breastfeed her baby, as the excretion in the milk of antithyroid drugs is very low. It is therefore safe to continue treatment and breastfeeding, provided not exceeding the doses [ 11–13].

Radioiodine therapy is contraindicated in pregnancy and breastfeeding, since it is taken up by the fetal/newborn thyroid and may result in thyroid ablation and hypothyroidism.

## Maternal evaluation

We evaluated the pregnant women by monitoring

- The weight: either weight loss (2 cases), or poor weight gain (5–8 kg)
- Persistent tachycardia was observed in 24 cases
- We also tested the levels of thyroid hormones and the levels of antibodies
- The blood pressure was normal in most of the cases

## Fetal evaluation

The fetus is at risk of thyroid dysfunction if the mother has TSH receptor antibodies and/or is taking anti–thyroid drugs during the third trimester.

Fetal evaluation (non stress test and ultrasound) was precisely performed and the fetal outcome was excellent. 

We performed serial ultrasound, before 28 and 32 weeks of gestation.

Fetal thyroid gland monitoring by ultrasonography is very important [14]. Demonstration of fetal goiter by ultrasonography is an important predictor of fetal thyroid dysfunction. It has been reported that fetal thyroid function is normal if there is no goiter, but fetal hyperthyroidism or hypothyroidism may be suspected if there is thyroid enlargement [14 – 16]. Thus the ultrasound became an extremely effective non–invasive tool for detection of fetal thyroid dysfunction. It also minimizes the necessity of invasive procedure to diagnose fetal thyroid dysfunction [13].

Fetal hypothyroidism is seen in mothers taking antithyroid drugs. Further fetal thyroid status evaluation may be obtained from an estimate of bone maturation at the distal femoral center and fetal heart rate as well as fetal growth. 

## Maternal–fetal complications

The complications met in our study were: abortion rate was 5 %. 

15% of pregnant women delivered prematurely

Maternal complications were rare (poor weight gain, tachycardia). 

Fetal complications included low birth weight (24%), fetal respiratory distress (10%)

## Conclusions

- Close teamwork between obstetricians and endocrinologists minimize fetal and maternal risks of Graves' disease, leading to good prognosis of both of them.
- First line therapy for Grave's disease during pregnancy includes antithyroid drugs (preferably propylthiouracil).
- To asses fetal thyroid function, fetal ultrasound at 28–32 weeks should be performed if there is evidence of active maternal Grave's disease.
- The improvement in the management of hyperthyroidism in pregnancy, particularly due to Graves' disease will depend on:
  - The capacity of the evaluation of thyroid function during gestation
  - Further elucidation of the immunology of Graves' disease focusing on the TSH receptor and its interaction with stimulating antibodies and artificial designer compounds, which will provide a rational immunologic therapy.
